# *Mcr* colistin resistance gene: a systematic review of current diagnostics and detection methods

**DOI:** 10.1002/mbo3.682

**Published:** 2018-07-04

**Authors:** John Osei Sekyere

**Affiliations:** ^1^ Department of Pharmaceutics Faculty of Pharmacy and Pharmaceutical Sciences Kwame Nkrumah University of Science and Technology Kumasi Ghana; ^2^ Department of Medical Microbiology Prinshof Medical School Campus Faculty of Health Sciences University of Pretoria Pretoria South Africa

**Keywords:** Colistin, detection methods, diagnostics, *mcr‐1*, polymyxin B

## Abstract

Resistance to colistin, mediated by chromosomal mutations and more recently, by plasmid‐borne *mcr* genes, is increasingly being reported in bacterial isolates taken from humans, animals, farms, foods, and the environment. To easily identify and contain this quickly spreading menace, efficient diagnostics that are cheaper, faster, simpler, sensitive, and specific have become indispensable and urgently necessary. A thorough and systematic review of the literature available at Pubmed, ScienceDirect and Web of Science was thus undertaken to identify articles describing novel and efficient colistin resistance‐ and *mcr* gene‐detecting methods. From the final 23 studies included in this review, both phenotypic and molecular tests were found. The phenotypic tests consisted of novel culture media viz., SuperPolymyxin™, CHROMagar COL‐*APSE* and LBJMR media, commercial automated MIC‐determining instruments such as MICRONAUT‐S, Vitek 2, BD Phoenix, Sensititre and MicroScan, and novel assays such as Colistin MAC test, Colispot, rapid polymxin NP test (RPNP), alteration of Zeta potential, modified RPNP test, MICRONAUT‐MIC Strip, MIC Test Strip, UMIC System, and Sensitest™ Colistin. Molecular diagnostics consisted of the CT103XL microarray, eazyplex^®^ SuperBug kit, and Taqman^®^/SYBR Green^®^ real‐time PCR assays, with 100% sensitivity and specificity plus a shorter turnaround time (<3 hr). Based on the sensitivity, specificity, cost, required skill and turnaround time, the RPNP test and/or novel culture media is recommended for under‐resourced laboratories while the Multiplex PCR or Taqman^®^/SYBR Green^®^ real‐time PCR assay alongside the RPNP or novel culture media is suggested for well‐resourced ones.

## 1 INTRODUCTION

The combined emergence of plasmid‐mediated carbapenem and colistin resistance, has thrown clinical medicine into a state of shock and therapeutic conundrum as infection control and management have become more difficult with little or no therapeutic options (Osei Sekyere, 2016; Osei Sekyere, & Asante, 2018; Osei Sekyere, Govinden, Bester, & Essack, 2016). Prior to the advent of carbapenem and colistin resistance in Gram‐negative bacteria, these two antibiotics were, respectively, used as last‐resort antibiotics for fatal and multidrug‐resistant infections (MDRIs) mediated by extended‐spectrum β‐lactamases (ESBLs) and carbapenemases (Osei Sekyere et al., 2016). Colistin and tigecycline alone, or in combination with an aminoglycoside, a fluoroquinolone, a carbapenem or fosfomycin, were used in managing carbapenem‐resistant infections before the emergence of colistin resistance (Osei Sekyere et al., 2016). However, the emergence of colistin resistance, particularly in carbapenem‐resistant strains, is compromising this combination regimen and has made tigecycline the sole last‐resort antibiotic for managing carbapenem‐ and colistin‐resistant MDRIs.

**Figure 1:**
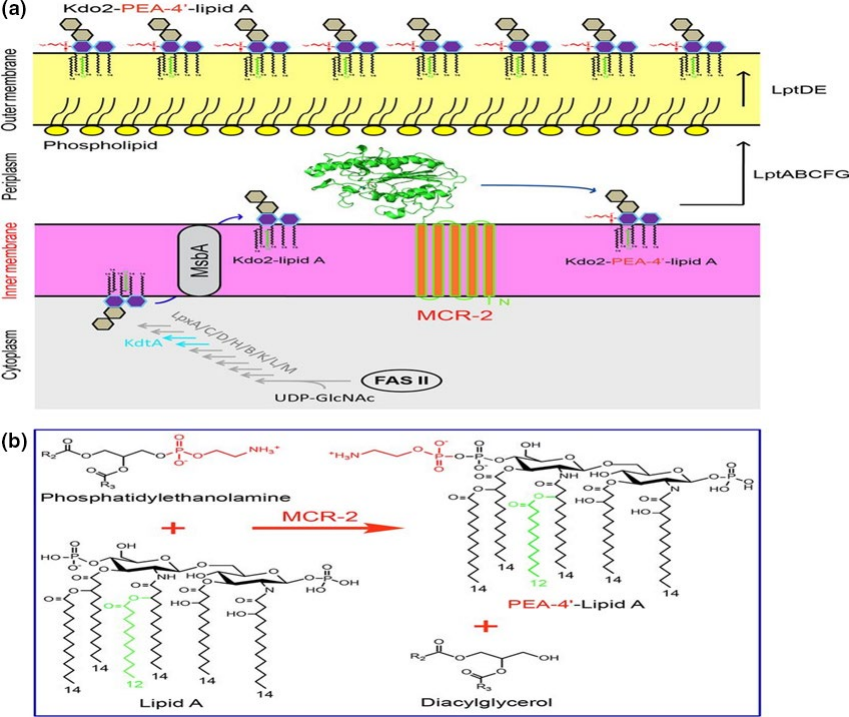
Mechanism of *mcr*‐mediated colistin resistance; adapted from (Sun et al., 2017). (a) Schematic representation for LPS‐lipid A modification by MCR‐2 in *E. coli*. In the cytoplasm, bacterial LPS‐lipid A is synthesized using UDP‐GlcNAc as the primer substrate. The fatty acid intermediates (C12 and C14) from the bacterial type II fatty acid synthesis (FAS II) pathway enter into the conservative 10‐step route of lipid A synthesis involving nine enzymes (LpxA, LpxC, LpxD, LpxH, LpxB, LpxK, LpxL, LpxM, and KdtA). The nascent lipid A from the cytoplasm is translocated by the ABC transporter MsbA, a lipid flippase (35), across the inner membrane into the periplasm. The integral membrane protein MCR‐2 is supposed to be localized on the periplasm side of inner membrane and catalyzes the chemical modification of the 2‐keto‐3‐deoxyoctulosonic acid (Kdo2)‐lipid A, giving Kdo2‐PEA‐4 = ‐lipid A. The modified form of Kdo2‐lipid A, Kdo2‐PEA‐4 = ‐lipid A, then is exported by LptABCFG and LptDE into the outer leaflet of the outer membrane (36), thus reducing the negative membrane charge. That is the reason for the low/decreased affinity of bacterial surface to the cationic antibiotic polymyxin. (b) Chemical reaction in which MCR‐2 catalyzes the modification of lipid A with 4 = ‐phosphatidylethanolamine. MCR‐2 catalyzes the addition of phosphatidylethano‐ lamine to position 4 =  of lipid A, giving the final products of both PEA‐4 = ‐lipid A and diacylglycerol

The emergence of carbapenem‐ and colistin‐resistance determinants in single strains ushers in a new age of pandrug resistance, and underscores the rapid depletion of our antimicrobial armamentarium (Delgado‐Blas, Ovejero, Abadia‐Patino, & Gonzalez‐Zorn, 2016; He et al., 2017; Sun et al., 2016; Yang et al., 2016). The detection of plasmids harboring both carbapenem and colistin resistance genes has made colistin resistance, which was hitherto solely mediated by mutations in and insertional inactivation of *phoPQ, ccrB, mgrB, pmrAB, lpxACD*, and *pmrHFIJKLM,* and restricted to chromosomal or vertical transmission, a grave threat to clinical medicine (Beceiro et al., 2014; Cannatelli et al., 2014; Giani et al., 2015; Jayol, Nordmann, Brink, et al., 2017; Jayol, Nordmann, Lehours, Poirel, & Dubois, 2017; Mavroidi et al., 2015; Wright et al., 2015). Chromosomal‐mediated colistin resistance through the above‐stated mutations and insertions, result in the addition of 4‐amino‐4‐deoxy‐L‐arabinose (L‐Ara‐4N), phosphoethaloamine (PEtN) or galactosamine moieties to the anionic phosphate groups at the 4′ or 1′ position of lipid A, which is the binding site of polymyxins, polypeptides that include colistin and polymyxin B (Esposito et al., 2017; Poirel, Jayol, & Nordmann, 2017) (Figure 1). The addition of any of these three molecules to lipid A reduces its anionic charges and prevents the cationic colistin from binding and initiating lysis and cell death (Abdul Momin et al., 2017; Poirel, Jayol, Nordmann, et al., 2017) (Figure 1).

**Figure 2:**
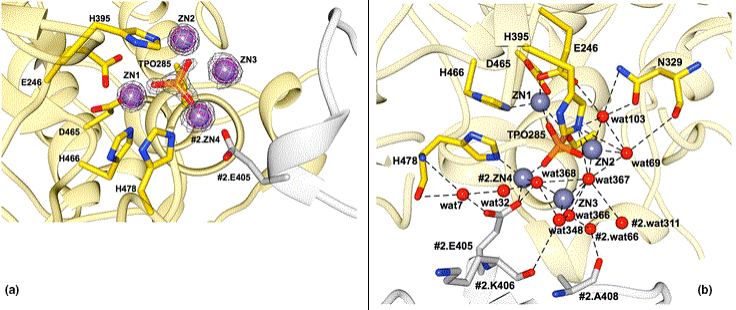
Catalytic domain structure of mcr‐1 enzyme; adapted from (Stojanoski et al., 2016). (a) Structure of the active‐site phosphothreonine with associated zinc ions. The phosphothreonine (TPO285) is represented as a yellow‐orange‐red stick model and the zinc ions (ZN1, ZN2, ZN3, and ZN4) that surround the phosphothreonine are shown as slate blue spheres. The 2Fo−Fc simulated annealing difference map of the final refined model contoured at σ = 4.0 is shown as a gray mesh. ZN4 is also coordinated by Glu405 from a neighboring molecule in the crystal. The neighboring MCR‐1 protein is colored white and labeled with the prefix #2. (b) Representation of the zinc ions identified in the active site of cMCR‐1. Zinc ions are shown as slate blue spheres and active‐site residues are represented in stick model. In yellow, is one MCR‐1 (#1) molecule, and in white, is another MCR‐1 (#2) molecule located adjacent to the first one. ZN4 from the second molecule is positioned at the interface and is shared by the two molecules. Structural water molecules are labeled and hydrogen bonds and zinc interactions are shown with dashed lines

The transferable plasmid‐mediated colistin resistance gene, *mcr*, and variants such as *mcr‐1.1, ‐1.2, 1.3, 1.4, 1.5, 1.6, 1.7* and *‐1.8* and *mcr‐2, ‐3, ‐4,* and *‐6*, is a PEtN transferase enzyme that enzymatically transfers PEtN to lipid A (Abdul Momin et al., 2017; Esposito et al., 2017; Poirel, Jayol, Nordmann, et al., 2017) (Figure 1). This, as described for the chromosomal mutations above, results in reduced anionic charges on lipid A, preventing electrostatic interactions with cationic polypeptide molecules such as polymyxins (colistin and polymyxin B), leading to colistin resistance (Esposito et al., 2017; Tendon, Poirel, & Nordmann, 2017). Addition of PEtN to the 4′ position of lipid A results in a compound called PEtN‐4′‐lipid A (PEA‐4′‐lipid A) (Figure 1). Interestingly, the catalytic domain of *mcr* enzymes resemble that of zinc metalloproteins. Consequently, PEtN can be inhibited by metal‐chelating agents such as dipicolinic acid (DA) and ethylene diamine tetra‐acetic acid (EDTA) (Figure 2) (Coppi et al., 2017; Esposito et al., 2017; Sun et al., 2017).

To stem the dissemination of colistin resistance, rapid, cheap and highly efficient diagnostics are especially crucial and urgently needed (Dona, Bernasconi, Kasraian, Tinguely, & Endimiani, 2017; Jayol, Nordmann, Brink, et al., 2017; Jayol, Nordmann, Lehours, et al., 2017). Effective infection control, infectious diseases’ management, and efficient diagnostics are inextricably intertwined as the former two cannot be accomplished without the latter. This has been demonstrated in the case of carbapenem resistance and *Candida auris* (Osei Sekyere, 2018; Osei Sekyere, Govinden, & Essack, 2015). To this end, several culture‐based polymyxin‐resistance screening media in the form of solid agar or broth, microarray, loop‐mediated isothermal amplification (LAMP), multiplex PCR, and real‐time PCR with either SYBR^™^ Green or Taqman^®^ probes have also been utilized (Abdul Momin et al., 2017; Carretto et al., 2018; Lescat, Poirel, & Nordmann, 2018; Matuschek, Åhman, Webster, & Kahlmeter, 2018; Rebelo et al., 2018). These diagnostics directly or indirectly detect polymyxin‐resistant Gram‐negative bacteria and/or *mcr‐1/‐2/‐3‐4/-5* producers from clinical or cultured bacterial specimen with varying turnaround times, sensitivities and specificities (Abdul Momin et al., 2017; Bernasconi et al., 2017; Chabou et al., 2016; Coppi et al., 2017; Rebelo et al., 2018).

The Clinical Laboratories Standard Institute (CLSI) and the European Committee on Antimicrobial Susceptibility Testing (EUCAST) have recommended the use of broth microdilution (BMD) as the standard testing protocol for determining colistin susceptibility among Gram‐negative bacteria (Chew, La, Lin, & Teo, 2017). However, the relatively higher skill and difficulty associated with integrating the BMD into normal clinical routines have made other recently developed culture media and assays very relieving (Jayol, Nordmann, Brink, et al., 2017; Jayol, Nordmann, Lehours, et al., 2017; Nordmann, Jayol, & Poirel, 2016a,b). Notwithstanding, the BMD is used as the gold standard in testing the essential agreement (EA), categorical agreement (CA), major error (ME), and very major error (VME) of colistin minimum inhibitory concentration (MIC)‐measuring diagnostics (Abdul Momin et al., 2017; Nordmann et al., 2016a,b; Poirel, Larpin et al., 2017). CA refers to agreement in the interpretation of the MIC between the test compared to BMD, and EA occurs when an MIC result is within a twofold dilution from the BMD's. A ME occurs when the tested MIC is resistant while the BMD MIC is susceptible. VMEs occur when the evaluated method's MIC was susceptible while BMD MIC was resistant (Chew et al., 2017).

Although there are studies evaluating the relative efficiencies of the various commercial susceptibility‐testing platforms and media, they are few and mostly undertaken with small sample sizes that do not express all known *mcr* types and variants (Jayol, Nordmann, Brink, et al., 2017; Jayol, Nordmann, Lehours, et al., 2017; Chew et al., 2017; Esposito et al., 2017; Carretto et al., 2018). As well, a standard and accepted protocol for screening, identifying and confirming colistin‐resistant Gram‐negative bacteria or *mcr‐*producing Enterobacteriaceae in clinical routines is nonexistent.

### 1.1 Purpose of this review

**Figure 3:**
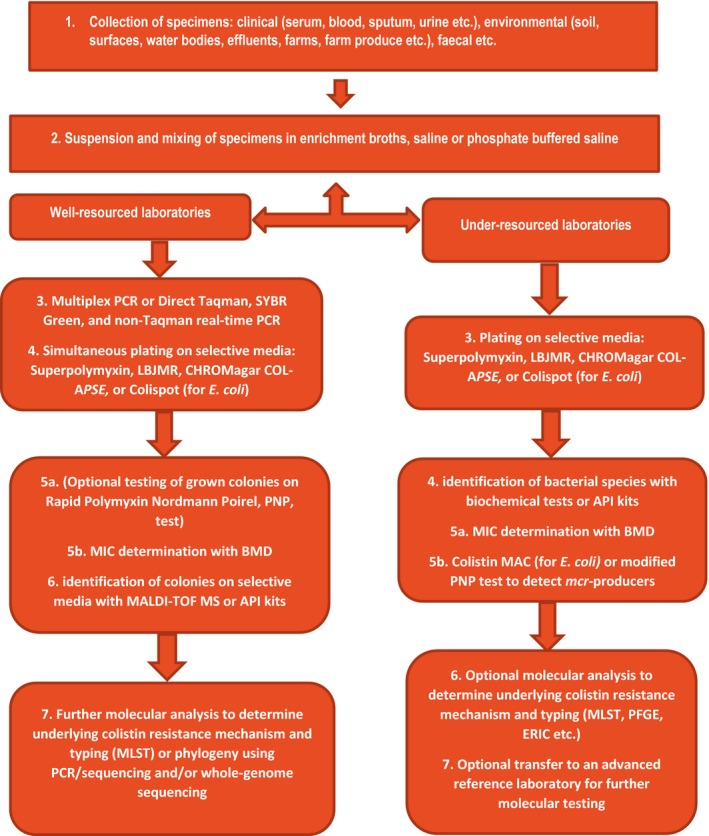
Flow diagram showing suggested screening and confirmation protocols for detecting polymyxin (colistin and polymyxin B)‐resistant bacteria in clinical microbiology laboratories

In the face of these challenges, this systematic review seeks to comprehensively describe all available polymyxin resistance‐ and *mcr‐*detecting diagnostics in the context of their composition (for culture media), primers and cycling conditions (for multiplex and real‐time PCR), sensitivities, specificities, turnaround time, skill, relative cost, EA, CA, ME, and VME. A flow diagram suggesting a standard protocol for screening, isolating, identifying and confirming colistin‐resistant isolates (Figure 3) is also included in this review, using the relative efficiencies, cost and required skill of the various diagnostics and detection methods as a guide. Finally, none of the 13 published reviews on *mcr‐1* and colistin resistance addresses colistin resistance‐ and *mcr‐*detecting diagnostics and detection methods, either narratively or systematically (Supplementary file S1), making this work the first, to my knowledge.

### 1.2 Methods used

**Figure 4:**
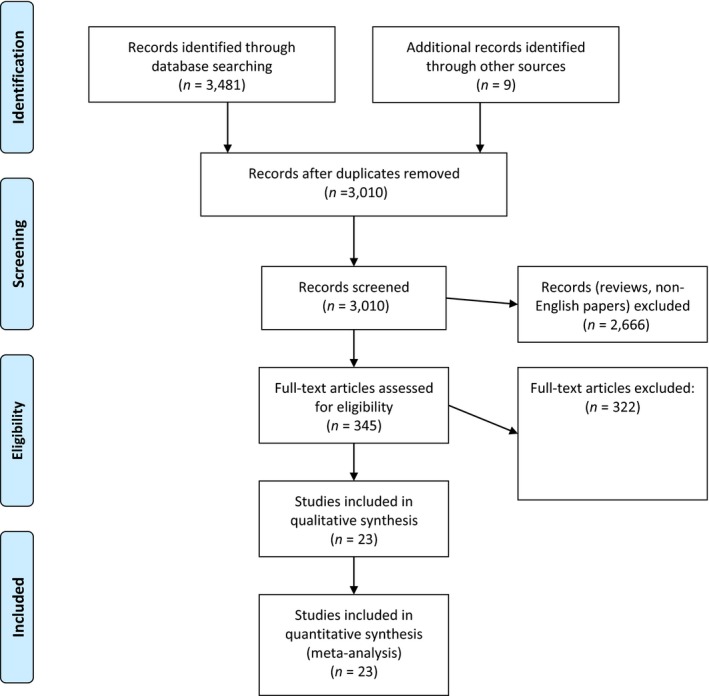
PRISMA‐ adapted flow diagram of included and excluded studies. Adapted from the PRISMA website (http://prisma‐ statement.org/PRISMAStatement/CitingAndUsingPRISMA.aspx) and article (Moher, Liberati, Tetzlaff, Altman, & Altman, 2009)

A systematic search of the literature was undertaken using the search words ‘*mcr‐1*′ and ‘colistin resistance’ on Pubmed, Science Direct and Web of Science on three different occasions. The dates filter was turned to between 2010 to May 20, 2018. All reviews, non‐English articles, and papers not describing diagnostic or detection methods were subsequently discarded. The PRISMA guidelines were followed in searching, screening and including papers for this review (Figure 4). The following data were extracted from the included articles: diagnostic tool or methods used, types and sample size of bacterial species used for the evaluation, sensitivity, specificity, EA, CA, ME, VME, turnaround time, media composition, real‐time PCR cycling conditions, cycle threshold, product size, primers and probes used, color of media, and appearance of colonies on media (Tables 1, 2, 3).

**Table 1:** Primers used in real‐time multiplex PCR for detecting the mcr gene

PCR type	Primer/probe	Amplified gene	Product size (bp)	Cycling conditions			Cycle threshold	Specimen types targeted		Reference (s)
Multiplex PCR	Mcr1_320bp_fw (AGTCCGTTTGTTCTTGTGGC), mcr1_320bp_rev (AGATCCTTGGTCTCGGCTTG), mcr2_700bp_fw (CAAGTGTGTTGGTCGCAGTT) mcr2_700bp_rev (TCTAGCCCGACAAGCATACC), mcr2_900bp_fw (AAATAAAAATTGTTCCGCTTATG) mcr3_900bp_rev (AATGGAGATCCCCGTTTTT) mcr4_1100bp_fw (TCACTTTCATCACTGCGTTG) mcr4_1100bp_rev (TTGGTCCATGACTACCAATG) MCR5_fw (ATGCGGTTGTCTGCATTTATC) MCR5_rev (TCATTGTGGTTGTCCTTTTCTG)	*MCR‐1* *MCR‐2* *Mcr‐3* *Mcr‐4* *Mcr‐5*	mcr‐1 (320 bp), mcr‐2 (715 bp), mcr‐3 (929 bp), mcr‐4 (1116 bp) mcr‐5 (1,644)	1 cycle of denaturation at 94°C for 15 min, 25 cycles of denaturation at 94°C for 30 sec, annealing at 58°C for 90 s and elongation at 72°C for 60 s, & final cycle of elongation at 72°C for 10 min. Amplification visualized on 1.5% agarose gel at 130 V & staining in ethidium‐bromide			Not applicable	Cultured Enterobacteriaceae species		Rebelo et al., (2018)
	mcr1‐mtpF [5′‐ATGCCAGTTTCTTTCGCGTG‐3′] and mcr1‐mtpR [5′‐ TCGGCAAATTGCGCTTTTGGC‐3′], mcr2‐mtpF [5′‐ GATGGCGGTCTATCCTGTAT‐3′] and mcr2‐mtpR [5′‐AAGGCTGACACCCCATGTCAT‐ 3′], mcr3‐mtpF [5′‐ACCAGTAAATCTGGTGGCGT‐3′] and mcr3‐mtpR [5′‐AGGACAACCTCGTCATAGCA‐3′], mcr4‐mtpF [5′‐TTGCAGACGCCCATGGAATA‐3′] and mcr4‐mtpR [5′‐GCCGCATGAGCTAGTATCGT‐ 3′], mcr5‐mtpF [5′‐GGACGCGACTCCCTAACTTC‐3′] and mcr4‐ mtpR [5′‐ACAACCAGTACGAGAGCACG‐3′]	*MCR‐1* *MCR‐2* *Mcr‐3* *Mcr‐4* *Mcr‐5*	mcr‐1 (502 bp), mcr‐2 (379 bp), mcr‐3 (296 bp), mcr‐4 (207 bp) mcr‐5 (608 bp)	denaturation at 94°C for 4 min; 30 cycles of 94°C for 5 s, 59°C for 20 s, and a single, final, elongation step at 72°C for 5 min. Elongation step was avoided as all PCR products <600 bp; electrophoresis in 2.5% agarose gel for 50 min			Not applicable	Cultured Enterobacteriaceae species		Lescat et al., (2018)
Real‐time PCR	mcr‐1_s(5′‐ATGGCACGGTCTATGATA‐3′), mcr‐1_FAM‐BHQ (5′‐CTACAGACCGACCAAGCCGA‐3′) and mcr‐1_as (5′‐CGG ATAATCCACCTTAACA‐3′)	*Mcr‐1*	155	Initial incubation: 15 min, 95°C; 45 cycles of 30 s at 95°C, 30 s at 55°C and 30 s at 72°C			20 and 27	Cultured bacteria, stool specimens, clinical samples		Nijhuis et al., (2016)
Real‐time PCR (Taqman®)	PE_F1(GCAGCATACTTCTGTGTGGTAC), PE_R1(ACAAAGCCGAGATTGTCCGCG), PE_Probe 1(6 FAM –GACCGCGACCGCCAATCTTACC‐TAMRA), PE_F2 (GGGTGTGCTACCAAGTTTGCTT), PE_R3 (TATGCACGCGAAAGAAACTGGC), PE_Probe (6 FAM –GCGCTGATTTTACTGCCTGTGGTG‐TAMRA)	*Mcr‐1*	145	Initial incubation: 15 min, 95°C; 35 cycles of 95°C for 30 s and 60°C for 1 min			18–25	Cultivated bacteria, chicken feces		Chabou et al., (2016)
Real‐time PCR (SYBR® Green)	mcr‐1‐FW(5′‐ACGCCATCTGCAACACCAA‐3′) and mcr‐1‐RV (5′‐ GCCAACGAGCATACCGACAT‐3′)	*Mcr‐1*	59	Incubation:50°C, 2 min for UNG; 1st denaturation: 95°C, 10 min; 2nd denaturation: 30/40 cycles (95°ºC, 15 s); annealing: 63°C, 10 s; extension (72°C, 10 s; Tm:78.4°C			34.37‐ ‐ >40 (native stools), 21–23 (enriched stools)	Stools/feces		Dona et al., (2017)
	mcr‐1‐qF1 (5′‐ACACTTATGGCACGGTCTATG‐3′) and mcr‐1‐qR1 (5′‐GCACACCCAAACCAATGATAC‐3′); mcr‐1‐qF2 (5′‐TGGCGTTCAGCAGTCATTAT‐3′) and mcr‐1‐qR2 (5′‐AGCTTACCCACCGAGTAGAT‐3′). mcr‐1‐F (5′‐ATGATGCAGCATACTTCTGTGTG‐3′) and mcr‐1‐R (5′‐TCAGCGGATGAATGCGGTGC‐3′).	*Mcr‐1*	120, 1646		95°C for 2 min and 40 cycles of 958Cfor 3 s,608°C for 20 s and 728°C for 7 s, followed by a ramp from728Cto 958C for melting analysis	NS			Cultured bacteria and stool	Bontron et al., (2016)
	mcr1‐qf (AAAGACGCGGTACAAGCAAC), mcr1‐qr (GCTGAACATACACGGCACAG), mcr2‐qf (CGACCAAGCCGAGTCTAAGG), mcr2‐qr (CAACTGCGACCAACACACTT), mcr3‐qf (ACCTCCAGCGTGAGATTGTTCCA), mcr3‐qr (GCGGTTTCACCAACGACCAGAA)	*Mcr‐1, mcr‐2, mcr‐3*	213 (*mcr‐1*), 92 (*mcr‐2*), 169 (*mcr‐3*)		A cycle of 50°C for 2 min, 95°C for 3 min, then 40 cycles of 95°C for 30 s, 60°C for 30 s, and 72°C for 30 s, followed by a ramp from72 to 95°C for melting curve stage	12.6∼16.7 (*mcr‐1*), 9.62 (*mcr‐2*), 13.3∼20.4 (*mcr‐3*)			cultured bacteria, feces and soil samples	Li et al., (2017)

**Table 2:** Composition, physical characteristics of screening media and phenotypic bacterial characteristics on media

Culture media	Color	Composition				Appearance of colonies	References
		Agar base	Chromogenic agent added	Antibiotics (mg/L)	Swarming inhibitors		
SuperPolymyxin™ media	Red	Eosin Methylene Blue (EMB)	No	Colistin sulfate (3.5) Daptomycin (10) Amphotericin B (5)	None	*E. coli*: characteristic metallic green/sheen. *Enterobacter* & *Klebsiella spp*.: brown, dark‐centred & mucoid colonies. Lactose fermenters: dark brown colonies Lactose non‐fermenters: colorless/light lavender	Bardet et al., (2017); Nordmann et al., (2016a,b)
CHROMagar COL‐APSE media	Pale‐cloudy appearance	Dehydrated CHROMagar (42.5 g/L), CHROMagar growth supplement S1 (2 ml), CHROMagar COL‐*APSE* supplement (4 ml)	Yes	Colistin sulfate oxazolidinones	p‐nitro‐ phenyl glycerol (PNPG)	*E. coli:* Dark‐pink to reddish. *Klebsiella, Enterobacter* & *Serratia spp*.: metallic blue. *Morganella spp*.: colorless natural pigmentation	Abdul Momin et al., (2017)
LBJMR media	Purple	Purple agar base (31 g/L) with glucose (7.5 g/L) and bromocresol purple	No	Colistin sulfate (4), vancomycin (50)	None	Enterobacteriaceae and *Enterococcus spp*.: yellow colonies	Bardet et al., (2017)

**Table 3:** Relative efficiencies of mcr diagnostics in detecting mcr‐1‐positive and polymyxin‐resistant Gram‐negative bacteria

Diagnostics	Species (n)	Sensitivity (%)	Specificity (%)	Relative cost	Relative skill required	Turnaround time (hr)	CA (%)^a^	EA (%)^a^	ME (%)^b^	VME (%)^c^	LOD(cfu/ml or reaction)^d^	References
Screening and culture‐based methods												
Broth microdilution (BMD)	Enterobacteriaceae (74)	71.4, 81.0	NS^e^	Cheap	Low	24	NA^f^	NA	NA	NA	NA	Chew et al., (2017)^g^
CHROMagar COL‐ *APSE*	Enterobacteriaceae (76); Gram‐negative non‐fermenters (6)	100	100	Cheap	Lowest	24	NA	NA	NA	NA	10^1^	Abdul Momin et al., (2017)
Colistin MAC test	Enterobacteriaceae (74)	100^h^	100^i^	Cheap	Lower	24	NA	NA	NA	NA	NS	Coppi et al., (2017)
Colispot	*Escherichia coli* (141)	100	100	Cheaper	Lowest	18–24	NA	NA	NA	NA	NA	Jouy et al., (2017)
Combined disc test ± EDTA (CDT)	Enterobacteriaceae (104)	96.7	89.6	Cheaper	Lowest	18–24	NA	NA	NA	NA	NA	Esposito et al., (2017)
Colistin MIC reduction (CMR) test	Enterobacteriaceae (104)	96.7	83.3	Cheaper	Lowest	18–24	NS	NS	NS	NS	NS	Esposito et al., (2017)
*E*‐test	Enterobacteriaceae (76, 32), *P. aeuginosa* (21), *Acinetobacter spp*. (22)	66.7, 76.2	NS	Expensive	Low	24	81.0‐85.0, 89.5, 92.1	47.0, 48.7–71.0, 75.0	1.9, 5.9, 5.13 (2/39)	12–26.1, 37.5 (13.5/36)	NS	Chew et al., (2017); Matuschek et al., (2018)
LBJMR media	Enterobacteriaceae (101); Gram‐negative non‐fermenters (17)	100	100	Cheap	Lowest	18–24	NS	NS	NS	NS	10	Bardet et al., (2017)
MIC Test strip® (MTM) (Liofilchem)	Enterobacteriaceae (32), *P. aeuginosa* (21), Acinetobacter spp. (22)	NS	NS	Expensive	Low	16–20	76.79	53.0–65.0	0	47.22 (17/36)	NS	Matuschek et al., 2018
MICRONAUT MIC‐Strip® (MERLIN Diagnostika)	Enterobacteriaceae (32), *P. aeuginosa* (21), *Acinetobacter spp*. (22)	NS	NS	Expensive	Low	16–20	91.0	99.0	12.82 (5/39)	5.56 (2/36)	NS	Matuschek et al., 2018
Rapid Polymyxin NP (RPNP) test	Enterobacteriaceae (70, 123, 200, 223)	100.0, 99.3, 98.7, 93.8,	100.0, 95.4, 94.9, 93.8,	Cheap	Low	<2	98.37	NA	2.5, 5.1	1.2	NS	Nordmann et al., (2016a,b); Jayol, Nordmann, Brink, et al., (2017); Jayol, Nordmann, Lehours, et al. (2017); Poirel, Larpin et al., (2017); Jayol, Kieffer et al., (2018)
Commercial RPNP	Enterobacteriaceae (223)	98.1	94.9	Expensive	Low	<3	NA	NA	5.1	1.9	NS	Jayol, Kieffer, et al., (2018)
Modified RPNP test	Enterobacteriaceae (104)	96.7	100.0	Cheap	Low	<2	NA	NA	NA	NA	NA	Esposito et al., (2017)
SensiTest™Colistin (Liofilchem)	Enterobacteriaceae (323, 32); Gram‐negative non‐fermenters (30, 43)	NS	NS	Expensive	Low	16–20	89.0, 98.9	88.0, 96.0	0.92, 17.95 (7/39)	1.46, 2.78 (1/36)	NS	Carretto et al., (2018); Matuschek et al., (2018)
SuperPolymyxin™	Enterobacteriaceae (68), Gram‐negative non‐fermenters (20)	86.0, 100.0	100.0	Cheap	Lowest	24–48	NA	NA	NA	NA	10^1^–10^2^	Abdul Momin et al., (2017); Bardet et al., (2017); Nordmann et al., (2016a,b)
UMIC (Biocentric)	Enterobacteriaceae (32), *P. aeuginosa* (21), *Acinetobacter* spp. (22), Gram‐negative bacilli (185)	NS	NS	Expensive	High	18–24	92.0, 91.9	82.0	7.69 (3/39), 0 (0/52)	8.33 (3/36), 11.3 (15/133)	NS	Jayol, Nordmann, et al. (2018); Matuschek et al., (2018)
Zeta potential (±EDTA) alteration	Enterobacteriaceae (104)	95.1	100.0	Very expensive	High	<1	NS	NS	NS	NS	NS	Esposito et al., (2017)
Automated commercial MIC testing platforms												
MICRONAUT‐S	Enterobacteriaceae (32), *P. aeuginosa* (21), Acinetobacter spp. (22)	NS	NS	Very expensive	High	18–24	89.0	96.0	15.38 (6/39)	5.56 (2/36)	NS	Matuschek et al., (2018)
MicroScan	Enterobacteriaceae (76), Gram‐negative bacilli (185)	100	NS	Very expensive	High	16–24	88.2, 91.9	NA	8.0, 26.9 (14/52)	4.0, 0.8 (1/133)	NS	Chew et al., (2017); Jayol, Nordmann, et al. (2018)
BD Phoenix/Phoenix 100™	Enterobacteriaceae (123, 323); Gram‐negative non‐fermenters (30)	91.87^j^	NS	Very expensive	High	16–24	96.8, 91.9	96.8, NS	0.46 (1/216), 0.0	2.74 (6/137), 12.5	NS	Jayol, Nordmann, Brink, et al., (2017); Jayol, Nordmann, Lehours, et al. (2017); Carretto et al., (2018)
Sensititre™	Enterobacteriaceae (76, 32), *P. aeuginosa* (21), *Acinetobacter spp*. (22), Gram‐negative bacilli (185)	95.2, 100	NS	Very expensive	High	18–24	>90, 95.0, 97.8	89.5,96.1–89.5, 96.0	11.8, 10.26 (4/39), 0 (0/52)	4.0, 0.0, 3.0 (4/133)	NS	Chew et al., (2017); Jayol, Nordmann, et al. (2018); Matuschek et al., (2018)
Vitek 2	Enterobacteriaceae (76)	95.2	NS	Very expensive	High	18–24	>90	93.4, 96.1‐93.4	0.0	36	NS	Chew et al., (2017)
Molecular methods												
Microarray (CT103XL)	Enterobacteriaceae (106)	100.0	100.0	Expensive	Higher	6.5	NA	NA	NA	NA	NA	Bernasconi et al., (2017)
Eazyplex® SuperBug (LAMP^k^)	Enterobacteriaceae (104)	100.0	100.0	Expensive	High	≤0.50	NA	NA	NA	NA	NS	Imirzalioglu et al., (2017)
Conventional PCR	Enterobacteriaceae (123,106, 104, 104, 84)	100.0	100.0	Very expensive	Higher	<3	NA	NA	NA	NA	NS	Imirzalioglu et al., (2017); Jayol, Nordmann, Brink, et al. (2017); Jayol, Nordmann, Lehours, et al. (2017); Bernasconi et al., (2017); Esposito et al., (2017); Abdul Momin et al., (2017)
Multiplex PCR	Enterobacteriaceae (49, 52)	100.0	100.0	Very expensive	Higher	<2–3	NA	NA	NA	NA	NS	Lescat et al., (2018); Rebelo et al., (2018)
Real‐time PCR	Enterobacteriaceae and non‐fermenters (87)	100.0	100.0	Very Expensive	Higher	<3	NA	NA	NA	NA	3–30	Nijhuis et al., (2016)
Real‐time PCR (Taqman®)	Enterobacteriaceae (80) and non‐fermenters (20)	100.0	100.0	Very Expensive	Higher	<2	NA	NA	NA	NA	10^1^–10^8^ DNA copies	Chabou et al., (2016)
Real‐time PCR (SYBR®‐Green)	Enterobacteriaceae (9)	100.0	100.0	Very Expensive	Higher	<3	NA	NA	NA	NA	10gDNA/reaction, 10^2,^ 1 mcr/10^6^ 16srRNA copies	Dona et al., (2017)
	Enterobacteriaceae (20)	100.0	100.0	Very Expensive	Higher	<3	NA	NA	NA	NA	10^2^ or 10^6^–10^2^ copies of mcr‐1	Bontron et al., (2016)
	Enterobacteriacea e (25)	100.0	100.0	Very Expensive	Higher	<3	NA	NA	NA	NA	10^2^, 1 mcr‐1/10^6^ 16S rRNA	Li et al., (2017)
Whole‐genome sequencing	Enterobacteriaceae (104,106)	100.0	100.0	Most Expensive	Highest	<48	NA	NA	NA	NA	NS	Bernasconi et al., (2017); Imirzalioglu et al., (2017); Rebelo et al., (2018)

Notes

a CA, categorical agreement.

b EA, essential agreement.

c ME, major error.

d VME, very major error.

e LOD, limit of detection.

f Not specified.

g Not applicable.

h All sensitivities and specificities are measured with respect to mcr‐1 while CA, EA, ME and VME are calculated with reference to BMD for both colistin and Polymyxin B.

i Except for *K. pneumoniae*.

Except for *K. pneumoniae*.

j Calculated from Jayol, Nordmann, André, Poirel, & Dubois (2018); Jayol, Kieffer et al., (2018) in which 10 colistin resistant were undetected by Phoenix BD system.

k Loop‐mediated Isothermal Amplification assay.

## 2 RESULTS AND DISCUSSION

A final list of 23 articles were included in this systematic review from the 3010 screened manuscripts (Figure 4). Of this number, three reported on the design and evaluation of novel culture media for screening polymyxin‐resistant Enterobacteriaceae and/or Gram‐negative non‐fermenters from cultured bacteria, clinical, fecal and environmental specimen: CHROMagar COL‐*APSE*, SuperPolymyxin™ and LBJMR media (Tables 2, 3). Eight studies reported on novel assays for either screening polymyxin (colistin)‐resistant Enterobacteriaceae and/or Gram‐negative nonfermenters, or for detecting *mcr‐*producing Gram‐negative bacteria from bacterial cultures: Colistin MAC test, Colispot, Combined Disc Test (CDT) ± EDTA test, Colistin MIC Reduction (CMR) ± EDTA test, Rapid Polymyxin Nordmann Poirel (RPNP) test, commercial RPNP test (ELITechGroup, Puteaux, France), Modified RPNP (MRPNP) test, and Zeta potential ± EDTA test (Tables 2, 3).

The MICRONAUT MIC‐Strip (MMS) (MERLIN Diagnostika Gmbh, Bornheim, Germany), the UMIC system (Biocentric, Bandol, France), the MIC Test Strip (MTS) (Liofilchem, Roseto degli Abruzzi, Italy) and Sensitest Colistin (Liofilchem, Roseto degli Abruzzi, Italy) (Carretto et al., 2018) are novel commercial but manual tests (Table 3). *E*‐test and BMD, which are older MIC‐determining methods, were evaluated by two and single studies, respectively (Table 3).

Commercial automated MIC‐determining equipment such as MICRONAUT‐S (MERLIN Diagnostika Gmbh, Bornheim, Germany), MicroScan, BD Phoenix (Becton Dickinson Diagnostics, USA), Sensititre (ThermoFisher Diagnostics) and Vitek II (BioMerieux) were also evaluated to determine their sensitivity, CA, EA, ME, and VME, using BMD as a gold standard (Table 3).

PCR and WGS were used in ≥2 molecular‐based studies (Table 3) as gold standards to evaluate the sensitivity and specificity of real‐time PCR, multiplex PCR, CT103XL micro‐array, and loop‐mediated amplification (LAMP) assays, which detected *mcr‐1/‐2/‐3‐/-4/‐5* resistance genes from bacterial cultures, clinical and fecal samples (Table 1). *Mcr‐1/‐2-*detecting assays were designed by single studies using micro‐array, real‐time PCR, and LAMP. Three studies designed real‐time SYBR^®^ Green PCR assays to identify *mcr‐1/‐2/‐3,* and two studies reported on a novel multiplex PCR assay to detect *mcr‐1/‐2/‐3/‐4/‐5* from culture (Tables 1, 3).

Thus, all the current colistin resistance‐determining diagnostics/assays can be grouped into phenotypic and molecular tests, in which the phenotypic tests, except in a few cases discussed below, mainly determines the presence or absence of colistin resistance in Enterobacteriaceae and nonfermenting Gram‐negative bacteria without establishing the underlying mechanism. On the other hand, all the molecular tests identified the presence of known *mcr* genes and variants, particularly *mcr‐1,* in Enterobacteriaceae without necessarily determining phenotypic colistin resistance (Rebelo et al., 2018). The various phenotypic and molecular colistin (polymyxin) resistance‐ and *mcr‐*detecting methods or assays described so far are comprehensively described below.

### 2.1 Phenotypic tests: Screening media and MIC‐determining tools

Simple and cheap (agar or broth) media that can easily but efficiently identify colistin‐resistant Gram‐negative bacteria while inhibiting nonresistant ones are crucial for surveillance purposes to early identify sources and carriers of these strains (Abdul Momin et al., 2017; Chew et al., 2017; Poirel, Joyal et al., 2017; Poirel, Larpin et al., 2017). But for the longer turnaround time of 18–24 hr required for these media, which is a major disadvantage, and lower *mcr* specificity and sensitivity compared to molecular methods, these screening media would be well‐suited for less‐resourced laboratories due to their lower costs and simple operating skill required (Table 3 and Figure 3). Screening media provides the advantage of quickly isolating colistin‐resistant bacterial strains from a matrix of components contained in environmental, foods, fecal and clinical specimens/samples. The isolated resistant strain can then undergo further identification, MIC and molecular analysis to fully characterize its species and determine its resistance mechanism (Abdul Momin et al., 2017; Bardet, Bardet, Page, Leangapichart, & Rolain, 2017; Nordmann et al., 2016a,b). Although molecular methods that can identify *mcr* genes in samples are available, they are more expensive, require advanced skill and in most cases, cannot detect resistant strains with novel resistance mechanisms or chromosomal mutations‐mediated resistance. Furthermore, the presence of *mcr* genes does not always translate phenotypically into colistin resistance (Abdul Momin et al., 2017; Bardet et al., 2017; Nordmann et al., 2016a,b). All published phenotypic tests that detect colistin resistance are described below, beginning from older methods to novel ones.

#### 2.1.1 MIC‐determiners: BMD, *E*‐test, UMIC, MMS, MTS, and automated commercial equipment

Determining the MIC of cultured bacterial strains isolated from various sources viz., environmental, clinical, fecal, and food, is a major routine step undertaken by microbiology laboratories to ascertain and confirm the resistance profile of an isolate. Tests and equipment that determine MICs use pure bacterial cultures and cannot undertake the testing directly on collected samples.

##### BMD

BMD is currently the accepted and gold standard for colistin MIC determination and evaluation of the EA, CA, ME, and VME of other MIC‐determining tools. Within the CLSI‐EUCAST joint declaration document espousing the BMD as the method of choice for determining MICs, it has been recommended that nonpolystyrene‐treated plates should be used (Jayol, Nordmann, Brink, et al., 2017; Jayol, Nordmann, Lehours, et al., 2017; Chew et al., 2017; Abdul Momin et al., 2017). This is because colistin can bind to or adsorb to plastics, thus reducing its concentration in the broth and ultimately affecting the MIC values; hence, colistin solutions should be stored in glass instead of plastics to maintain accurate concentrations (Jayol, Nordmann, Brink, et al., 2017; Jayol, Nordmann, Lehours, et al., 2017; Chew et al., 2017; Abdul Momin et al., 2017). Furthermore, it is recommended that sulfate salts of colistin instead of the colistimethate, which is used in human medicine, should be used in determining colistin MICs without adding polysorbate 80 (Jayol, Nordmann, Brink, et al., 2017; Jayol, Nordmann, Lehours, et al., 2017; Chew et al., 2017; Abdul Momin et al., 2017). However, agar dilution and disc diffusion methods were ruled out by the joint CLSI‐EUCAST document because the larger molecular size of polymyxins (colistin) makes it poorly diffusible through agar (Abdul Momin et al., 2017; Chew et al., 2017). Hence, comparing BMD MIC results with other methods such as the agar dilution, *E*‐test and other agar‐based antimicrobial sensitivity tests (AST) should be done with caution.

EUCAST recommends the following colistin breakpoints for Enterobacteriaceae, *Pseudomonas aeruginosa,* and *Acinetobacter spp*.: susceptible ≤2 mg/L; resistant >2 mg/L. CLSI has colistin breakpoints for *Pseudomonas aeruginosa,* and *Acinetobacter spp*. but not for Enterobacteriaceae: susceptible ≤2 mg/L, intermediate = 4 mg/L, and resistant ≥8 mg/L (Clinical and Laboratory Standards Institute (CLSI), 2017). However, CLSI has an epidemiological cut‐off value of 2 mg/L for *Escherichia coli, Klebsiella pneumoniae, Raoultella ornithinolytica, Enterobacter aerogenes*, and *Enterobacter cloacae* that define isolates as either wild type or nonwild type (Chew et al., 2017; Vasoo, 2017).

A single study evaluated the ability of BMD to detect MCR‐1‐positive Enterobacteriaceae where sensitivity to colistin and polymyxin B was, respectively, 71.4% and 81.0% at a breakpoint of ≤2 mg/L, or 90.5% and 85.7% at a cut‐off of ≤1 mg/L (Chew et al., 2017) (Table 3). This study found that the MICs of polymyxin B and colistin were not interchangeable, although the results from selective culture media supplemented with either polymyxin B or colistin were found to be the same (Bardet et al., 2017; Nordmann et al., 2016a,b; Poirel, Larpin et al., 2017). It is thus obvious that BMD results cannot be relied on completely to detect *mcr‐*positive isolates. This is not surprising as *mcr‐1‐*positive strains have been found to be susceptible to colistin, and acquired colistin resistance is known to confer low‐level colistin resistance (Chew et al., 2017). On the other hand, it suggests that decreasing the colistin MIC cut‐off to ≤1 mg/L can increase the sensitivity of BMD and other commercial MIC‐determining platforms to detect *mcr‐*positive isolates (Chew et al., 2017). Thus, it is necessary to confirm *mcr* expression with molecular assays as BMD is not 100% MCR‐sensitive.

Both the clinical significance of colistin‐susceptible *mcr‐*positive strains and a correlation between MICs and clinical outcome, as a guide to treatment, are still not well established. In addition, the clinical effect of heteroresistance is still unknown, especially when colistin is given as combination therapy (Chew et al., 2017). Heteroresistance, the phenomenon of having a mixed population of colistin‐resistant and ‐susceptible strains or a population of strains with different levels of colistin resistance, is one of the challenges confronting MIC determination and interpretation as repeated testing of such strains yields different results (Jayol, Nordmann, Brink, et al., 2017; Jayol, Nordmann, Lehours, et al., 2017; Chew et al., 2017; Abdul Momin et al., 2017). This is especially pronounced in *Enterobacter spp*. in which heteroresistance has been associated with the *hsp*60 (heat shock protein) gene; strains from hsp60 clusters I, II, IV, VII, IX, X, XI, and XII are usually hetero‐resistant while those of cluster III, V, VI, VIII, and XIII are typically susceptible (Chew et al., 2017). Thus, *hsp*60 sequencing could aid in colistin AST. However, the MICs of a wide range of isolates need to be tested to improve on the detection of heteroresistance (Chew et al., 2017). Chew et al. (2017) have thus proposed that an intermediate colistin breakpoint is introduced in the interim for Enterobacteriaceae, as is the case for *P. aeruginosa* and *Acinetobacter spp*. by the CLSI (Clinical and Laboratory Standards Institute (CLSI), 2017), until a better correlation between *mcr* genes and colistin resistance is established.

The BMD method however, cannot be adopted in routine clinical microbiology laboratories without some challenges. The method is seen by many as laborious and time consuming as it requires the manual preparation of antibiotic solutions, broths, etc., besides the 24‐hr incubation time required to read results. The weighing of the powders requires precision, without which errors could be introduced (Jayol, Nordmann, Brink, et al., 2017; Jayol, Nordmann, Lehours, et al., 2017; Poirel, Larpin et al., 2017). These challenges make the other automated methods, none of which meets the CLSI's recommended performance standards for commercial AST systems (EA ≥ 90%, CA ≥ 90%, VME ≤ 1.5%, ME ≤ 3.0%) (Chew et al., 2017), and recently developed selective screening media that cannot determine MICs, more welcome and easily patronized (Nordmann et al., 2016a,b; Jayol, Nordmann, Brink, et al., 2017; Jayol, Nordmann, Lehours, et al., 2017).

##### *E*‐test

Colistin *E*‐test strips were evaluated against BMD and found to have the poorest EA (75%) as well as the highest VME (12%) after Vitek 2 (36%) (Table 3) (Chew et al., 2017). The poorer efficiency of *E*‐test is not surprising given the poor diffusibility of colistin through agar, which made the CLSI‐EUCAST joint committee to rule out *E*‐test, agar dilution, and disc diffusion for colistin AST (Jayol, Nordmann, Brink, et al., 2017; Jayol, Nordmann, Lehours, et al., 2017).

##### UMIC, MMS, and MTS

The UMIC, MICRONAUT MIC‐Strip (MMS), and MIC Test Strip (MTS) are all commercial but manual MIC‐determining tests. The UMIC and MMS diagnostics are similar in that they both consist of a 12‐well plastic strip containing up to 11 different colistin concentrations for testing a single isolate while the MTS is a gradient‐based test composed of a paper strip with increasing colistin concentrations. The MTS is like *E*‐test where the colistin diffuses through solid agar media on which it is placed while the UMIC and MMS use broth akin to BMD. Due to its diffusion‐based approach, the MTS was found to have more VMEs (16‐18/43) than BMD‐based tests, albeit it performed slightly better than BMD‐based tests among colistin‐susceptible isolates (MIC ≤ 2 mg/L) (Matuschek et al., 2018). Although UMIC and MMS are cheaper and easier to use, the automated BMD platforms performed better (Table 3) (Jayol, Nordmann, et al., 2018; Matuschek et al., 2018). Particularly, UMIC failed to detect low MIC colistin‐resistant isolates with intrinsic and acquired resistance as well as four *Stenotrophomonas maltophilia* isolates with higher MICs (Jayol, Nordmann, et al., 2018).

##### Automated commercial systems: MicroScan, MICRONAUT‐S, BD Phoenix, Sensititre and Vitek 2

Four studies have evaluated the efficiency of automated commercial MIC‐determining systems,that is, MicroScan, Sensititre, MICRONAUT‐S, BD Phoenix (Phoenix 100^™^) and Vitek 2 (Table 3) (Jayol, Nordmann, Brink, et al., 2017; Jayol, Nordmann, Lehours, et al., 2017; Carretto et al., 2018; Jayol, Nordmann, et al., 2018; Matuschek et al., 2018). The MicroScan and Sensititre are the only platforms that recorded 100% sensitivity in detecting *mcr‐*producing Enterobacteriaceae, but Sensititre had higher CA (90.1%–97.8%), and lower ME (0%–11.8%) than that of MicroScan (88.2%, and 15.4%–26.9%, respectively). The MicroScan had a higher ME of 15.8% (Table 3). The MICRONAUT‐S system was shown to have the same EA as Sensititre, albeit it had lower CA (89%) and higher ME (15.38%) and VME (5.56%) (Table 3)(Matuschek et al., 2018). It can be observed from Table 3 that Vitek had the poorest sensitivity (42.9%) to *mcr‐1* producers and highest VME (36%) among all the commercial systems, which has also been confirmed by BioMerieux (Carretto et al., 2018). Moreover, it has been already reported that the Vitek 2 has poor sensitivity in detecting heteroresistance (Jayol, Nordmann, Brink, et al., 2017; Jayol, Nordmann, Lehours, et al., 2017). A study using the agar dilution reference method to evaluate MicroScan found a CA of 87.3% while another using the BMD supplemented with polysorbate 80 to evaluate Sensititre found no VMEs for Enterobacteriaceae and two MEs for *P. aeruginosa* (Chew et al., 2017).

Thus, in terms of sensitivity, EA, CA, ME, and VME, Sensititre has so far been the most efficient followed by the BD Phoenix (Phoenix 100™), MICRONAUT‐S and MicroScan. The BD Phoenix and Phoenix 100™ instruments, whose results are interpreted by the BD Epicenter Software, have lower sensitivity for the *mcr* gene. Moreover, strains with colistin MICs as high as 16–128 mg/L were not determined by the BD Phoenix instrument, which was possibly due to heteroresistance in those strains (Jayol, Nordmann, Brink, et al., 2017; Jayol, Nordmann, Lehours, et al., 2017). Further testing with larger samples comprising of most members of the Enterobacteriaceae and Gram‐negative nonfermenters expressing all known colistin resistance mechanisms is necessary to confirm these preliminary findings, which were obtained with smaller sample sizes and nonrepresentative intrinsic and acquired colistin resistance isolates.

#### 2.1.2 Novel assays: Chelator‐based and non‐chelator‐based tests

Novel assays involving the use of cation‐adjusted Mueller–Hinton broth (CAMHB) and agar (CAMHA) with or without colistin and/or metal chelators such as EDTA and DA supplementation, have been designed and evaluated to assess their ability to detect colistin‐resistant and *mcr‐*producing Enterobacteriaceae. Such novel assays include the colistin MAC test, Colispot, CDT, CMR, RPNP, commercial RPNP, MRPNP, Sensitest™ Colistin (STC) and alteration of Zeta potential tests (Table 3). Many of these novel assays, specifically the colistin MAC test, CDT, CMR, MRPNP, and alteration of Zeta potential, though phenotypic, detects MCR*‐*positive bacteria from cultures using EDTA or DA to chelate zinc and inhibit the enzymatic activity of MCR‐1 (Esposito et al., 2017). Through the inhibition of MCR‐1 activity because of zinc chelation, the MCR‐1‐positive isolate is unable to maintain its resistance to colistin. Notably, the chelator‐based MCR‐detecting tests were more efficient with *E. coli* than with other species such as *K. pneumoniae* (Coppi et al., 2017; Esposito et al., 2017). The remaining assays, which does not involve metal chelators, mainly detects colistin resistance*,* albeit they had very high MCR‐1 sensitivity (Table 3). These assays are comprehensively described below.

##### 2.1.2.1 Rapid Polymyxin NP test (RPNP) and commercial RPNP

The RPNP test is one of the novel colistin resistance‐determining assays designed by Nordmann et al. (2016a,b)and evaluated with 200 isolates of global origin expressing diverse colistin resistance mechanisms. It had a sensitivity and specificity of 99.3% and 95.4%, respectively, albeit latter evaluation studies recorded between 93.8% and 100% and 95.4% and 100% sensitivity and specificity values, respectively (Table 3). The test has a short turnaround‐time of ≤2 hr and is relatively easier and cheaper to perform as it requires simple reagents for preparing two solutions: a polymyxin stock solution and a rapid polymyxin NP solution that can be prepared extemporaneously or stored at −4°C or −20°C for a year (Nordmann et al., 2016a,b; Jayol, Nordmann, Brink, et al., 2017; Jayol, Nordmann, Lehours, et al., 2017). A microtiter plate with wells labeled A1‐A4 and B1‐B4, containing colistin‐free solutions (A1‐A4), rapid polymyxin solution and colistin (B1‐B4), 0.85% NaCl (A1 and B1), colistin‐susceptible negative‐control strain (A2 and B2), colistin‐resistant positive‐control strain (A3 and B3), and the test isolate (A4 and B4), was used for the test. A color change from orange to yellow in the test and positive‐control strains’ wells with no color change in the negative‐control and NaCl‐containing wells was interpreted as colistin resistant and vice versa, that is, the absence of a color change (orange to yellow) in all but the positive‐control well was interpreted as colistin‐susceptible (Nordmann et al., 2016a,b).

The RPNP has now been commercialized by the ElitechGroup (Puteaux, France), and was comparable to the noncommercial RPNP in turnaround time and efficiency (1.9% VME, 5.1% ME, 98.1% sensitivity and 94.9% specificity) except that the former had a slightly better VME (1.2%). Using 223 enterobacterial isolates, both tests had better sensitivity for heteroresistant isolates, including three heteroresistant *Enterobacter cloacae* isolates; additional tests to confirm this property is necessary (Table 3) (Jayol, Kieffer, et al., 2018).

Bacterial colonies isolated from acidifying media such as Drigalski, McConkey or bromocresol purple agar resulted in more false‐positive results while those from Mueller–Hinton agar (MHA), Luria‐Bertani agar (LBA), Columbia agar +5% sheep blood, chocolate agar, Uriselect 4 agar, and eosin methylene blue (EMB) agar provided accurate results with this test. The faster turnaround time, relative simplicity, higher efficiency, and lower cost of this method makes it one of the best colistin resistance, including MCR‐producers, diagnostic assay available.

##### Colistin MAC test: A DA chelator‐based mcr‐detecting test

Coppi et al. (2017) designed and evaluated the only BMD‐DA‐based *mcr‐*detecting assay using a fixed concentration of 900 μg/ml DA and increasing concentrations of colistin (0.125–8 μg/ml) (Coppi et al., 2017). The test uses the BMD method with slight modification, in terms of DA supplementation, to screen for MCR*‐*producers. A cut‐of ≥8‐fold increase in MIC was interpreted as *mcr‐*positive while an MIC reduction of ≤2‐fold was interpreted as *mcr‐*negative. Repeated testing of isolates with intermediate results (having MIC fold‐changes between mcr‐positive and mcr‐negative: 3–7 MIC fold changes) found them to be resistant; however, isolates that remain intermediate upon repeated testing are interpreted as indeterminate. The test was evaluated with 74 clinical (*n* = 70), environmental (*n* = 1) and laboratory (*n* = 3) Enterobacteriaceae strains and was 100% sensitive and specific for *E. coli, Citrobacter spp*. and *Enterobacter spp*.; however, it could not detect two *mcr‐*positive *K. pneumoniae*. Thus, this test needs further testing with more Enterobacteriaceae species and sample size, including more *K. pneumoniae* isolates to assess its efficiency beyond *E. coli, Citrobacter spp*., and *Enterobacter spp*.

The authors used DA instead of EDTA because DA has a higher affinity for zinc. Moreover, when the authors used the disc diffusion method instead of the BMD with DA, they could not attain the same 100% sensitivity and specificity (Coppi et al., 2017). This is obviously due to the poorer diffusion of colistin through agar as already stated above. Similarly, Esposito et al. (2017) showed that the addition of EDTA to colistin discs yielded lower sensitivity and specificity, 96.7% and 89.6%, respectively, for mcr detection (Esposito et al., 2017). Hence, disc diffusion‐based tests with either DA or EDTA as a means to detect *mcr‐*positive enterobacteria is not advised. This test has a turnaround time of 24 hr due to incubation.

##### EDTA‐based assays: CDT, CMR, MRPNP, and alteration of Zeta potential

Esposito et al. (2017) designed four EDTA‐based assays to detect MCR‐positive *E. coli* isolates , which was evaluated using 109 Enterobacteriaceae isolates from humans, animals, and food. The four tests, CDT, CMR, MRPNP, and alteration of Zeta potential, are based on the chelation of zinc ions, which are necessary for the enzymatic activity of the PEtN transferase, MCR‐1.

The CDT test uses two 10 μg colistin discs placed on CAMHA plates swabbed with the test isolates as described for the disc diffusion testing by CLSI/EUCAST (EUCAST, 2013; Clinical and Laboratory Standards Institute (CLSI), 2015). One of the two discs was impregnated with 10 μl of 100 mmol/L EDTA prior to incubation. An incremental difference of ≥3 mm between the colistin‐impregnated disc and the colistin‐EDTA‐impregnated disc was interpreted as MCR‐1*‐*positive *E. coli*; however, this cut‐off resulted in five false positives. The CDT was 96.7% and 89.6% sensitive and specific, respectively, which makes this method less efficient.

The CMR test was designed according to the EUCAST‐recommended BMD method (Esposito et al., 2017; EUCAST, 2013), with the addition or nonaddition of 80μg/ml EDTA to wells containing 0.06–32 μg/ml (or to 512 μg/ml for intrinsic‐resistant strains) colistin. However, CAMHB was not used in this assay as calcium and magnesium supplementation will chelate with EDTA and affect the test's outcome. In addition, calcium has been found to enhance the activity of putative PEtN transferases in *E. coli* (Esposito et al., 2017). Furthermore, 0.5 McFarland's concentration of isolates diluted to 1:100 were used in the CMR test. A ≥ 4‐fold colistin MIC reduction in EDTA‐containing wells was interpreted as MCR‐1‐positive; nevertheless, false positives and negatives were recorded with this cut‐off. The MCR‐1 sensitivity and specificity of this method was, respectively, 96.7% and 83.3%, which makes it less efficient than the CDT method and more sensitive than BMD; it is thus inadvisable to use this test. Given the laborious nature of this test vis‐a‐vis that of the CDT, the CDT is more recommendable than the CMR assay.

The RPNP test was modified into the MRPNP test by the addition of two extra wells containing 80 μg/ml EDTA (without colistin) and 80 μg/ml EDTA plus 5 μg/ml colistin (Esposito et al., 2017). All colistin‐resistant isolates were positive for the PNP test, that is, changed color from orange to yellow, but only MCR‐1‐positive isolates were inhibited by EDTA, in that EDTA‐containing wells resulted in no color change (orange) after incubating for 1–4 hr (Esposito et al., 2017). Thus, isolates with positive PNP results that showed no color change (orange) in the presence of EDTA were interpreted as MCR‐1‐positive while those with positive PNP results and color change (yellow) in the presence of EDTA were MCR‐1 negative (Esposito et al., 2017). The sensitivity and specificity of this test was, respectively, 96.7% and 100.0% with a turnaround‐time of <4 hr, which makes it better than the CDT and CMR tests in terms of time and efficiency in detecting MCR‐1‐positive *E. coli*.

The alteration of Zeta potential test bases on the resultant surface‐membrane ionic charges of *E. coli* in the absence and presence of 80 μg/ml EDTA to detect MCR‐1‐producers. A ZetaPALS ZetaPotential Analyzer (Brookhaven Instruments Corporation, Holtsville, NY) was used to measure the particle size (diameter, mm) and Zeta potential (mV) of the bacterial cells. Colistin‐susceptible isolates had greater anionic surface charges of between −21.54 and −44.21 mV while colistin‐resistant ones had lesser anionic surface charges of ≤−20 mV (−4.20 to −19.34 mV) due to the presence of PEtN, L‐Ara4N or galactosamine on lipid A. Thus, MCR*‐*1*‐*positive strains had lower Zeta potential by virtue of the substitution of lipid A with PEtN‐4′‐lipid A (Esposito et al., 2017). A Zeta potential ratio, Rzp (Rzp = ZP_+EDTA_/ZP_‐EDTA_), was calculated from the Zeta potential recorded in the presence (ZP_+EDTA_) and absence (ZP_‐EDTA_) of EDTA and used as a measure of the presence or absence of MCR‐1. A Rzp cut‐of value of ≥2.5 was interpreted as MCR‐1 positive; however, a false‐negative result was obtained, possibly due to lower or no MCR‐1 expression in that isolate (Esposito et al., 2017). The test had a sensitivity and specificity of 95.1% and 100.0%, respectively (Esposito et al., 2017), which makes it second to only the MRPNP test in terms of efficiency among the EDTA‐based assays.

This study, for the first time, confirmed that colistin resistance resulted from reduction in surface anionic charges, which reduced colistin's binding affinity for lipid A (Esposito et al., 2017). It also showed that EDTA increased the anionic charges on the surface membrane of MCR‐1‐positive isolates such that the Zeta potential of MCR‐1‐positive strains became similar to that of colistin‐susceptible ones. Thus, further Zeta potential alteration tests with a larger sample size and representation of all colistin resistance mechanisms is needed to confirm these findings as this test can be easily adopted and used in many well‐resourced microbiology laboratories.

##### Colispot

Jouy et al. (2017) designed a novel phenotypic method, inspired by a technique designed for detecting defensins, to identify colistin‐resistant and MCR‐positive *E. coli*. A total of 141 (an initial 106 and an additional 35) *E. coli* from animal fecal samples were used in evaluating the assay, which involved the dropping of 10 μl of 8 mg/L colistin solution onto a Mueller–Hinton plate swabbed with 0.5 McFarland concentration of the test isolate. After 18–24 hr incubation, the isolate with >5 mm clear inhibition zone, that is, with no colony within the inhibition zone, was interpreted as colistin‐susceptible while those with colonies growing within the inhibition zone were interpreted as colistin resistant. The inhibition zone size was dependent on the colistin concentration used. The drops were placed on the plates in a manner that allowed at least a 2 cm distance between the centers of the drops. The 8 mg/L colistin concentration resulted in about 10 mm inhibition zones and clearly distinguished between colistin‐resistant and ‐susceptible strains (Jouy et al., 2017). The test was thus highly efficient in detecting colistin‐resistant *E. coli* and can be adopted in human medicine due to its simplicity and low cost.

##### Sensitest™ Colistin (STC)

STC (Liofilchem, Italy) is a new commercial test kit for determining colistin MICs for four isolates at a time. It comes with lyophilized colistin in seven‐two‐fold dilutions (0.25–16 μg/ml), with one additional well as growth control (Carretto et al., 2018). The test is similar to the BMD testing, albeit much simpler and limited to only colistin testing for a maximum of four test isolates. Following the manufacturer's as well as EUCAST and CLSI instructions, Carretto et al. (2018) evaluated the STC kit with 353 bacterial isolates and found it to have an EA of 96%, a CA of 98.9%, an ME of 0.92% and a VME of 1.46% (Table 3). Notably, an EA of 98.8% was recorded for MCR‐1‐positive isolates. However, a study by EUCAST with 75 isolates showed that STC had an EA of 88% with seven VMEs and one ME (Carretto et al., 2018). Thus, further evaluations with more isolates expressing diverse colistin resistance mechanisms is necessary to establish the relative efficiency of this kit in detecting colistin resistance.

The MIC results were read visually using turbidity, pinpoint colonies (*Hafnia alvei*) or buttons at the bottom of the wells. They also found the kit to be highly stable, reliable, and reproducible even at room temperature and varying temperatures. It was found to be highly reproducible (Carretto et al., 2018). Further, minimum bactericidal concentration could be also determined from the STC plate by spotting 1–10 μl from the wells unto CAMHA plates.

#### 2.1.3 Novel agar‐based screening media: SuperPolymyxin™, CHROMagar COL‐*APSE* and LBJMR media

There are currently three novel polymyxin (colistin/polymyxin B) resistance‐detecting screening media (Tables 2 and 3): SuperPolymyxin,™, CHROMagar COL‐*APSE* and LBJMR media. The major drawback to these media is their longer turnaround time (18–48 hr) for detecting colistin‐resistant Gram‐negative bacteria as well as their inability to confirm the presence of MCR‐producers. However, they can preliminarily detect all colistin‐resistant isolates in addition to those with novel colistin‐resistance mechanisms (Abdul Momin et al., 2017; Bardet et al., 2017; Nordmann et al., 2016a,b). These screening media commonly contain lower concentrations of colistin to inhibit susceptible strains and detect isolates with acquired colistin resistance, daptomycin/vancomycin to inhibit Gram‐positive bacteria, and amphotericin B to inhibit fungal growth. Some of them contain chromogenic compounds for species differentiation, but they all prevent swarming by *Proteus spp*. (Table 2). The components, sensitivity, specificity and limit of detection (LOD) of these media are described below.

##### SuperPolymyxin^™^

SuperPolymyxin, which is now marketed as a commercial patented product by ELITech Group solutions (www.elitechgroup.com/product/Superpolymyxin/) as SuperPolymyxin™, is the first screening medium developed to detect both intrinsic and acquired polymyxin‐resistant Enterobacteriaceae isolates from clinical, environmental, food and fecal specimen (Nordmann et al., 2016a,b). Higher colistin concentrations and presence of deoxycholates in earlier media inhibited strains with acquired resistance (MIC of 4–8 mg/L) due to lower colistin‐resistance levels (Nordmann et al., 2016a,b; Bardet et al., 2017). Hence, the SuperPolmyxin was developed with 3.5 μg/ml colistin, EMB powder to selectively inhibit non‐Gram‐negative bacteria, 10 μg/ml daptomycin (because vancomycin potentiated colistin's activity against several Gram‐negative bacteria) and 5 μg/ml amphotericin B (Table 2). The stock solutions used for preparing the media could be stored at −20°C for a year (Nordmann et al., 2016a,b). The medium was evaluated with 88 Gram‐negative bacteria and resulted in a sensitivity, specificity and LOD of 100%, 100% and 10^1^ (10^1−2^ for spiked stool samples) cfu/ml, respectively.

Besides *P. aeruginosa, Stenotrophomonas maltophilia* and the intrinsically resistant *Burkholderia spp*. that grew between 24 and 48 hr, the remaining resistant test isolates grew on the media within 24 hr; hence, SuperPolymyxin is more sensitive to Enterobacteriaceae than to nonfermenters. The EMB differentiated the species for easy identification (Table 3): lactose fermenters were dark brown colonies, *E. coli* had a characteristic metallic sheen, etc. Moreover, a low‐inoculum *K. pneumoniae* mixed with heavier inoculum of *Proteus mirabilis* was easily differentiated by SuperPolymyxin. The medium also inhibited *Candida albicans, Staphylococcus aureus* and colistin‐susceptible *E. coli* for at least 7 days at 4°C (Nordmann et al., 2016a,b). These qualities have made SuperPolymyxin one of the most patronized colistin‐resistance screening media.

##### CHROMagar COL‐APSE

The second colistin‐resistance agar‐based screening medium to be developed after the SuperPolymyxin was the CHROMagar COL‐*APSE* media that also detects colistin‐resistance in Enterobacteriaceae and Gram‐negative nonfermenting bacteria (Abdul Momin et al., 2017). A major advantage of CHROMagar COL‐*APSE* over SuperPolymyxin is the former's ability to identify Gram‐negative non‐fermenters more efficiently (Abdul Momin et al., 2017). The medium's composition (shown in Table 2), is such as to prevent swarming of *Proteus spp*. and improve upon the differentiation and identification of more species viz., dark‐pink to reddish (*E. coli*), metallic blue (*Klebsiella, Enterobacter* and *Serratia spp*.) etc. while *E. coli* is the only identifiable species on SuperPolymyxin. Evaluation of the media with 84 isolates resulted in an LOD of 10^1^cfu/mL just as SuperPolymyxin except that SuperPolymyxin could not grow a strain of *Acinetobacter baumannii* (MIC = 8 mg/L) and a strain of *S. maltophilia* (MIC = 32 mg/L). This could be due to synergy between daptomycin and colistin (Abdul Momin et al., 2017). Moreover, SuperPolymyxin was found to support only 86% (50/58) of MCR‐1‐positive *E. coli*, suggesting a lower MCR‐1 sensitivity than CHROMagar COL‐*APSE;* however, further evaluation tests are necessary to confirm this finding. Notably, the SuperPolymyxin was better at suppressing colistin‐susceptible *Salmonella spp*.

Additional studies evaluating these two media are lacking and further tests are necessary to show the media with the best colistin‐resistance and MCR‐detecting efficiency, particularly in identifying heteroresistant strains. It is, however, clear that the CHROMagar COL‐*APSE* has a broader target spectrum than the SuperPolymyxin.

##### LBJMR

The Lucie‐Bardet‐Jean‐Marc‐Rolain medium is the most recent colistin‐resistance screening medium to be developed for identifying colistin‐resistant Enterobacteriaceae and Gram‐negative nonfermenters as well as vancomycin‐resistant *Enterococci* (VRE) from cultured bacteria and stool samples (Bardet et al., 2017). Preliminary tests showed that purple agar base with glucose and bromocresol purple (Table 2) provided better results (sensitivity and specificity) than other combinations and media such as BD Cepacia medium and Columbia Colistin Nalidixic Acid agar+5% sheep blood (Bardet et al., 2017). Evaluation with 143 cultured bacterial isolates and 68 stool samples, followed by screening of 1052 stool samples from around the world, resulted in 100% sensitivity and specificity, with an LOD of 10^1^. The LBJMR medium inhibited *Proteus spp*. swarming 48 hr after incubation, and was as sensitive as SuperPolymyxin in detecting MCR‐positive bacteria from stool samples and culture; however, it was more sensitive than SuperPolymyxin in identifying non‐fermenters. Both Enterobacteriaceae and *Enterococci* appear as yellow colonies on the medium's purple background, but with different colony sizes (Bardet et al., 2017).

While the combination of daptomycin and colistin on EMB agar led to systematic inhibition of MCR‐positive *E. coli,* particularly those with lower MICs, the addition of amphotericin B, vancomycin or daptomycin to LBJMR medium did not affect its sensitivity (Bardet et al., 2017). Rather, LBJMR medium detected low concentrations of pathogens in cystic fibrosis samples, including *Burkholderia cepacia,* than Cepacia medium (Bardet et al., 2017). Direct culturing of samples without prior decontamination is possible on the LBJMR medium. Primary cultures can also be directly analyzed, using PCR and AST on LBJMR without subculturing (Bardet et al., 2017). This medium still requires further multicentre studies and comparative evaluation with the SuperPolymyxin and CHROMagar‐COL‐*APSE* media. However, its efficiency in detecting both colistin‐ and vancomycin‐resistant bacteria is very welcoming due to the clinical importance of colistin and vancomycin resistance.

### 2.2 Molecular diagnostics

But for their cost and higher skill requirements, the shorter turnaround time and higher efficiency (100% sensitivity and specificity) of molecular diagnostics in detecting MCR‐producing Enterobacteriaceae and *mcr* genes at very low concentrations in cultured bacteria as well as in clinical, fecal, environmental and food samples make them ideal tools (Tables 1 and 3). Microarray, LAMP, multiplex PCR and real‐time PCR assays have so far been designed to directly or indirectly identify *mcr* genes in Enterobacteriaceae (Tables 1 and 3). These assays cannot confirm colistin resistance as colistin susceptible MCR‐positive strains exist (Chew et al., 2017). Moreover, they cannot detect unknown colistin‐resistance mechanisms due to their dependence on highly specific primers and probes; only WGS can identify unknown colistin resistance mechanisms (Osei Sekyere & Asante, 2018).

#### 2.2.1 Microarray

Bernasconi et al. (2017) evaluated a new commercial microarray that could simultaneously detect both β‐lactamases and *mcr‐1/‐2* genes from bacterial culture. Using 106 Enterobacteriaceae strains of human, animals, and environmental sources, the CT103XL microarray, which uses a multiple‐ligation detection reaction, could identify all isolates expressing ESBLs, *mcr‐1* and *mcr‐2* genes, including variants such as *mcr‐1.1, mcr‐1.2, mcr‐1.3* up to *mcr‐1.7,* within 6.5 hr at a cost of ~€ 50: sensitivity and specificity was 100% (Bernasconi et al., 2017). However, the CT103XL could not detect *mcr‐3* genes, which has a 45% and 47% sequence homology to *mcr‐1* and *mcr‐2,* respectively. Other *mcr*‐expressing species, besides *E. coli*, should be also tested on this instrument to ascertain its ability to identify *mcr‐1/‐2* in diverse enterobacterial species. Each probe of the microarray consists of two arms with a universal primer binding site, target‐specific gene sequence and a zip code for the hybridization. Ligated and amplified probes were hybridized to the microarray, visualized with biotin‐labeled primers and interpreted automatically with software (Bernasconi et al., 2017). A major advantage to the microarray diagnostic technology is its potential to be upgraded with new or emerging resistance genes to detect more *mcr* gene types and variants. However, the cost and skilled involved will make it inaccessible to under‐resourced laboratories.

#### 2.2.2 Loop‐mediated isothermal amplification

A commercially available LAMP instrument called eazyplex^®^ SuperBug (Amplex Biosystems GmbH, Giessen, Germany) that detects *mcr‐1* from cultured bacteria with a turnaround time of ≤30 min was evaluated by Imirzalioglu et al. (2017) with 104 Enterobacteriaceae isolates: 67 were MCR‐positive, 37 were MCR‐negative and nine were intrinsically resistant to colistin (Imirzalioglu et al., 2017). The kit was 100% sensitive and specific. The sample preparation was simple and can be used on the field (livestock farms, food processing plants or hospitals) for diagnostic purposes as the Genie^®^ II instrument is mobile and can last for 4 hr without power supply (Imirzalioglu et al., 2017). However, the kit's ability to directly detect *mcr‐1* from samples without preculture has not been assessed and it can only process six samples per hour; the additional cost for scaling up for more samples are unknown (Imirzalioglu et al., 2017). Given the mobility, efficiency and shorter turnaround time of this kit, it will be very advantageous to improve upon it by increasing the spectrum of *mcr* types and variants it can detect as well as enhance its ability to directly detect these genes in samples without culturing.

#### 2.2.3 Conventional, multiplex, & real‐time PCR, and WGS

Conventional PCR and WGS are the gold‐standards and first diagnostic tools used in identifying the *mcr‐1* gene from swine *E. coli* isolates (Liu et al., 2016; Rebelo et al., 2018). While conventional PCR can only detect known *mcr* resistance genes within a shorter period, WGS can identify all known or unknown colistin resistance mechanisms within at most 2 days depending on the WGS instrument used (Osei Sekyere & Asante, 2018). In addition, conventional PCR can only identify one *mcr* type per reaction while WGS can identify all colistin resistance mechanisms per single reaction (Osei Sekyere & Asante, 2018; Xavier et al., 2016). To enable the multiple detection of several *mcr* genes and variants in a single reaction, seven studies have designed real‐time and multiplex PCR assays using either Taqman^®^ probes, self‐designed probes or SYBR^®^ Green (Tables 1 and 3).

Nijhuis et al. (2016) were the first to design a real‐time PCR‐based *mcr‐1* detection assay using self‐designed primers and probes (Table 1) (Nijhuis et al., 2016). In that study, they used 87 isolates (only 26 were *mcr‐1‐*positive and all were in *E. coli*) from poultry, calves and humans and found the assay to be 100% sensitive and specific, with an LOD of 3–30 cfu/reaction (Table 1). The assay could directly detect *mcr‐1* from stool samples. However, no *mcr‐1* was found in the other species viz., *Klebsiella, Enterobacter, Pseudomonas, Vibrio, Salmonella, Aeromonas*, and *Acinetobacter spp* (Nijhuis et al., 2016). Thus, the ability of the assay to detect *mcr‐1* in other species besides *E. coli* should be evaluated.

Chabou et al. (2016) also designed a quantitative Taqman^®^ PCR assay, published immediately after that of Nijhuis et al. (2016) *,* to quantitatively and qualitatively detect *mcr‐1* genes in cultured bacteria and chicken feces. Using a total of 100 bacterial isolates from humans and animals, of which only 18 were *mcr‐1‐*positive (*E. coli *= 12, *K. pneumoniae *= 6), and 833 broiler fecal samples having five *mcr‐1*‐positive strains, the assay proved to be 100% sensitive and specific with an LOD of 10^1^–10^8^ DNA copies (Tables 1 and 3). This assay could directly identify *mcr‐1* genes from biological samples with high specificity due to the Taqman^®^ probes used. As explained above, the number and species diversity of the MCR‐1‐harboring strains used in this study were nonrepresentative. Hence, the assay needs to be subjected to further evaluations with larger *mcr‐*containing samples and species.

Three SYBR^®^ Green‐based real‐time PCR assays have so far been designed to detect *mcr‐1, mcr‐2* and/or *mcr‐3* in Enterobacteriaceae. Bontron, Poirel, and Nordmann (2016) first designed a SYBR^®^ Green‐based real‐time PCR to detect only *mcr‐1* from cultured bacteria and directly from human and stool samples (Tables 1 and 3) (Bontron et al., 2016). Only eight of the 19 isolates used in this study were *mcr‐1‐*positive. The assay was 100% sensitive and specific, with an LOD of 10^2^ cultured bacteria. This study thus needs further evaluation with a larger sample size and different enterobacterial species.

Dona et al. (2017) also designed a SYBR^®^ Green real‐time assay to identify *mcr‐1* from fecal and cultured samples (Dona et al., 2017). However, they had to first suspend the fecal samples in Luria Bertani (LB) enrichment broth overnight followed by subsequent plating on selective agar plates supplemented with 4 mg/L colistin to get 100% sensitivity and specificity. Using native stools directly without an enrichment step resulted in lower sensitivity, which could be due to the presence of PCR inhibitors, inadequate or few *mcr‐1‐*producing strains and/or the negative effect of long‐term storage (at −80°C without a cryoprotectant) of stool samples (Dona et al., 2017). The assay was also used to test DNA directly extracted from native and enriched stool samples, with native nonenriched stool samples resulting in higher cycle thresholds (Ct) of between 34.37 and >40 while enriched stools resulted in lower *C*t values of 21–23. A total of 88 stool samples from volunteers were used in evaluating this assay, which had an LOD of 10^1^ DNA copies/reaction (Dona et al., 2017). Evidently, the enrichment step used in this assay substantially increased the sensitivity and reduced the Ct value of this assay and could be adopted in other assays. However, the enrichment step takes at least 12 hr, making it time‐consuming. The assay's sensitivity in directly determining *mcr‐1* in human stools is thus limited, without an enrichment step, compared to other molecular tests.

Another SYBR^®^ Green‐based real‐time assay was designed by Li et al. (2017) to detect *mcr‐1, mcr‐2*, and *mcr‐3* genes in Enterobacteriaceae from clinical, soil, and fecal specimen. This is the second molecular real‐time PCR assay designed to detect *mcr‐3* genes. The test was evaluated with 25 isolates plus *mcr‐1, ‐2*, and *‐3‐*containing mutants, resulting in 100% sensitivity and specificity, with an LOD of 10^2^ cultured bacteria. A copy of *mcr‐1* gene per 10^−6^ 16S rRNA copies could be detected. However, the assay could not detect all three *mcr* genes in a single reaction as is obtainable from a Taqman assay (Li et al., 2017). Further developments in this assay to increase the number of *mcr* gene types that can be detected and enable it to detect all *mcr* genes in a single reaction will make it one of the best molecular diagnostic tool available.

Two most recent molecular assays that can detect *mcr‐1, ‐2, ‐3, ‐4* and *‐5* genes were designed by Rebelo et al. (2018) and Lescat et al. (2018) using a multiplex PCR, agarose gel (1.5% and 2.5%, respectively) electrophoresis and ethidium bromide staining (Lescat et al., 2018; Rebelo et al., 2018). Although Rebelo et al.'s assay was designed to screen for *mcr* genes in *E. coli* and *Salmonella spp*. from animals (calves and pigs) in Europe, it can be extended to humans as a screening agent in well‐resourced laboratories. The assays were 100% sensitive and specific, and, respectively, found *mcr‐1* and *mcr‐4, mcr‐1* and *mcr‐3,* as well as *mcr‐1* and *mcr‐5* genes in single *E. coli* and *Salmonella enterica* isolates in a single reaction, meaning that the assays can identify single and multiple *mcr* genes in single strains. Lescat et al. (2018)'s method had a shorter turnaround time (<2 hrs) and used internal controls, which were absent in that of Rebelo et al. (2018) (Lescat et al., 2018; Rebelo et al., 2018). An isolate with an MIC of 2 mg/L was found to harbor an *mcr‐1* gene, which suggests the need to revise the epidemiological cut‐off for colistin in Enterobacteriaceae as well as introduce an intermediate resistance as suggested by Chew et al. (2017). This is necessary to identify MCR‐positive but susceptible strains that will otherwise not be detected.

## 3 CONCLUSION

The rapid expansion and dissemination of the *mcr* gene across bacterial species and regional boundaries is a major cause for concern, underscoring the urgency for better, simpler and cheaper diagnostic tools that can quickly and effectively detect colistin‐resistant bacteria. Among the currently available diagnostic tools, the RPNP test, which has a turnaround time of ≤2 hr, and/or the LBJMR, SuperPolymyxin, or CHROMagar COL‐*APSE* medium will be ideal for under‐resourced laboratories due to their lower cost as initial screening tools. This can be followed up with the colistin MAC or MRPNP tests, which, respectively, have turnaround times of 24 hr and <2 hr, to identify *mcr* producers. Colistin‐resistant strains can be sent to well‐resourced laboratories for further molecular tests if necessary (Figure 4). For well‐resourced laboratories, the multiplex PCR assay, the Taqman or SYBR Green real‐time PCR assays can be directly used on cultures or on biological and environmental samples alongside the RPNP test and/or LBJMR, SuperPolymyxin, or CHROMagar COL‐*APSE* medium to simultaneously identify *mcr* producers and colistin‐resistant isolates. Further species identification and typing can be undertaken with an API kit or MALDI‐TOF MS and PCR or WGS, respectively (Figure 4).

Going forward, further evaluations and modifications of available tests and methods should be undertaken to improve on the sensitivity, specificity, turnaround time, and costs. Moreover, periodic surveillance of hospitals, farms, foods, and the environment should be undertaken to quickly detect and contain colistin‐resistant and *mcr‐*producing strains from further dissemination. This is necessary to obtain the true prevalence of colistin resistance and *mcr* genes, and inform colistin stewardship, treatment guidelines, and protocols for colistin‐resistant infections.

## CONFLICT OF INTEREST

The author has no conflict of interest or transparency to declare.
