# Supplementary material for: Mcr colistin resistance gene: a systematic review of current diagnostics and detection methods
Source: Microbiologyopen. 2018 Jul 4;8(4):e00682. doi: 10.1002/mbo3.682 (PMC6530528; doi:10.1002/mbo3.682)
Supplement: Supplementary file 1 [file MBO3-8-e00682-s001.pdf]

List of published reviews on colistin resistance and mcr-1

[1] Leangapichart T, Rolain J-M, Memish ZA, Al-Tawfiq JA, Gautret P. Emergence of drug resistant bacteria at the Hajj: A systematic review. Travel Med Infect Dis 2017;18:3–17. doi:10.1016/j.tmaid.2017.06.008.

[2] Logue CM. Comprehensive resistome analysis reveals the prevalence of NDM and mcr-1 in Chinese poultry production 2017:1–5. doi:10.21037/jlpm.2017.06.02.

[3] Rhouma M, Fairbrother JM, Beaudry F, Letellier A. Post weaning diarrhea in pigs: risk factors and non-colistin-based control strategies. Acta Vet Scand 2017;59:31. doi:10.1186/s13028-017-0299-7.

[4] Rhouma M, Letellier A. Extended-spectrum β-lactamases, carbapenemases and the mcr-1 gene: is there a historical link? Int J Antimicrob Agents 2017;49:269–71. doi:10.1016/j.ijantimicag.2016.11.026.

[5] Alonso CA, Zarazaga M, Ben Sallem R, Jouini A, Ben Slama K, Torres C. Antibiotic resistance in Escherichia coli in husbandry animals: the African perspective. Lett Appl Microbiol 2017;64:318–34. doi:10.1111/lam.12724.

[6] Giamarellou H. Epidemiology of infections caused by polymyxin-resistant pathogens. Int J Antimicrob Agents 2016;48:614–21. doi:10.1016/j.ijantimicag.2016.09.025.

[7] Hsu L-Y, Apisarnthanarak A, Khan E, Suwantarat N, Ghafur A, Tambyah PA. Carbapenem-Resistant Acinetobacter baumannii and Enterobacteriaceae in South and Southeast Asia. Clin Microbiol Rev 2017;30:1–22. doi:10.1128/CMR.00042-16.

[8] Poirel L, Jayol A, Nordmann P. Polymyxins: Antibacterial Activity, Susceptibility Testing, and Resistance Mechanisms Encoded by Plasmids or Chromosomes. Clin Microbiol Rev 2017;30:557–96. doi:10.1128/CMR.00064-16.

[9] Al-Tawfiq JA, Laxminarayan R, Mendelson M. How should we respond to the emergence of plasmid-mediated colistin resistance in humans and animals? Int J Infect Dis 2017;54:77–84. doi:10.1016/j.ijid.2016.11.415.

[10] Teo JQM, Cai Y, Lim T-P, Tan TT, Kwa AL-H. Carbapenem Resistance in Gram-Negative Bacteria: The Not-So-Little Problem in the Little Red Dot. Microorganisms 2016;4. doi:10.3390/microorganisms4010013.

[11] Bakthavatchalam YD, Pragasam AK, Biswas I, Veeraraghavan B. Polymyxin susceptibility testing, interpretative breakpoints and resistance mechanism: an update. J Glob Antimicrob Resist 2017. doi:10.1016/j.jgar.2017.09.011.

[12] Rhouma M, Beaudry F, Theriault W, Letellier A. Colistin in Pig Production: Chemistry, Mechanism of Antibacterial Action, Microbial Resistance Emergence, and One Health Perspectives. Front Microbiol 2016;7:1789. doi:10.3389/fmicb.2016.01789.

[13] Schwarz S, Johnson AP. Transferable resistance to colistin: a new but old threat. J Antimicrob Chemother 2016;71:2066–70. doi:10.1093/jac/dkw274.
